# Evaluation of the prevalence of adolescent scoliosis and its associated factors in Gansu Province, China: a cross-sectional study

**DOI:** 10.3389/fpubh.2024.1381773

**Published:** 2024-07-30

**Authors:** Jin Huang, Haitao Zhang, Jiantao Wen, Lili Liu, Shihong Xu, Xingsheng Wang, Chen Zhang, Huaming Wang, Shengtai Pei, Xiaojuan Cui, Juan Wang, Dezhi Tang, Jun Zhao

**Affiliations:** ^1^Longhua Hospital Shanghai University of Traditional Chinese Medicine, Shanghai, China; ^2^Gansu Provincial Hospital of Traditional Chinese Medicine, Lanzhou, Gansu, China; ^3^Shanghai University of Traditional Chinese Medicine, Shanghai, China

**Keywords:** scoliosis, adolescence, risk factors, prevalence, cross-sectional study

## Abstract

**Introduction:**

Gansu Province is situated in the northwest region of China, characterized by diverse and complex topography and a rich diversity of ethnic groups. This study aims to explore the prevalence and risk factors of adolescent suspected scoliosis in Gansu Province through a cross-sectional population study.

**Methods:**

From April 2022 to July 2022, a prospective cross-sectional study was conducted in Baiyin City, Jinchang City, Lanzhou City, Linxia Hui Autonomous Prefecture, and Gannan Tibetan Autonomous Prefecture in Gansu Province. The screening covered 3,118 middle and high school students across 24 institutions, including middle and high schools. Diagnosis of suspected scoliosis was established through visual inspection, the Adams forward bend test, and measurement of trunk rotation angle. Employing a custom-designed questionnaire, demographic data were collected, and the prevalence of suspected scoliosis was calculated. Univariate and multivariate logistic regression analyses were employed to assess factors associated with suspected scoliosis.

**Results:**

A total of 3,044 participants were ultimately included in the analysis. The overall prevalence of suspected scoliosis was 5.68% in Gansu Province. The peak prevalence for boy is at 14 years (6.70%), while for girl, it is at 15 years (8.75%). Lanzhou City exhibits the highest prevalence rates (boy, 9.82%; girl, 10.16). The results of univariate logistic regression analysis presented that BMI (OR = 0.92, 95% CI: 0.88–0.96), altitude of habitation (1,600 m-2000 m) (OR = 0.50, 95% CI: 0.34–0.73), altitude of habitation (2000 m-3321 m) (OR = 0.58, 95% CI: 0.40–0.83), family medical history (OR = 1.56, 95% CI: 1.02–2.31), and shoulders of unequal height (OR = 1.49, 95% CI: 1.09–2.03) were significantly correlated with suspected scoliosis. The multivariate logistic regression analysis indicated that BMI (OR = 0.91, 95% CI: 0.86–0.95), altitude of habitation (1,600 m-2000 m) (OR = 0.35, 95% CI: 0.23–0.54), altitude of habitation (2000 m-3321 m) (OR = 0.39, 95% CI: 0.24–0.60), family medical history (OR = 1.66, 95% CI:1.08–2.49), and shoulders of unequal height (OR = 1.45, 95% CI:1.06–1.99) were independently associated with suspected scoliosis.

**Conclusion:**

Low BMI, residence at an altitude of 1,600 m-3321 m, family medical history, and shoulders of unequal height were independently associated with an increased prevalence of suspected scoliosis. It is recommended to promptly screen high-risk adolescents for suspected scoliosis, provide effective preventive and intervention measures.

## Introduction

Scoliosis refers to a spinal deformity with a Cobb angle >10° on standing coronal X-rays (1). The various types of scoliosis encompass idiopathic, congenital, neuromuscular, syndromic, and other etiologies leading to spinal deformities. Idiopathic scoliosis (IS) emerges as the predominant subtype, constituting roughly 80% of cases, particularly prevalent among adolescents aged 10–18 (2). According to epidemiological studies, the prevalence of scoliosis varies from 0.20 to 3.10% in several countries globally (3–6). A cross-sectional study involving 99,695 children in mainland China reported an overall scoliosis prevalence of 5.14% (7). In recent years, there has been a continuous rise in the incidence of scoliosis in China due to increased academic pressure and reduced physical activity and sleep time among primary and secondary school students (8).

Scoliosis exerts a substantive influence on the well-being of youthful individuals. As the Cobb angle escalates, it may precipitate complications such as back pain (9), aberrant cardiopulmonary function (10), sleep disorders (11), and profound deformities. These ramifications can impinge upon the psychological well-being of adolescents, impeding their seamless integration into routine social activities, culminating in disability (12). Scoliosis is a condition caused by the combined influence of multiple factors. Current mainstream research identifies common risk factors for scoliosis, including low BMI, gender differences, ethnic disparities, asymmetrical backpack carrying, low physical activity, and prolonged screen time (13–16). Nevertheless, despite the identification of numerous contributing factors to scoliosis, the comprehensive pathogenesis and natural progression of scoliosis continue to elude complete understanding. Furthermore, most studies on adolescent scoliosis in China have focused on the southeastern regions, and there is limited epidemiological evidence regarding scoliosis in the northwestern regions of China.

Gansu Province is a representative region with diverse topography in Northwest China, covering a total area of 454,000 square kilometers and a population of 26.17 million. The terrain in Gansu Province is complex and varied, including mountains, plateaus, plains, river valleys, deserts, and gobi, providing a comprehensive landscape. Additionally, Gansu Province is characterized by its multi-ethnic composition, with 44 ethnic minorities. Given its distinctive topography and diverse ethnic composition, Gansu Province emerges as a pivotal locale for conducting epidemiological studies on adolescent scoliosis, addressing the gap in research on adolescent scoliosis in this region.

By identifying high-risk factors potentially associated with adolescent idiopathic scoliosis, targeted screening can significantly enhance screening efficiency and accuracy, facilitating the development of personalized prevention and intervention plans. Increasing screening frequency and healthcare services in high-risk regions not only reduces healthcare costs but also optimizes equitable allocation of healthcare resources.

Therefore, this study aims to explore the prevalence and risk factors of adolescent scoliosis in Gansu Province through a cross-sectional population study, offering valuable insights for the development of preventive measures.

## Materials and methods

### Study design and data sources

**Figure 1:**
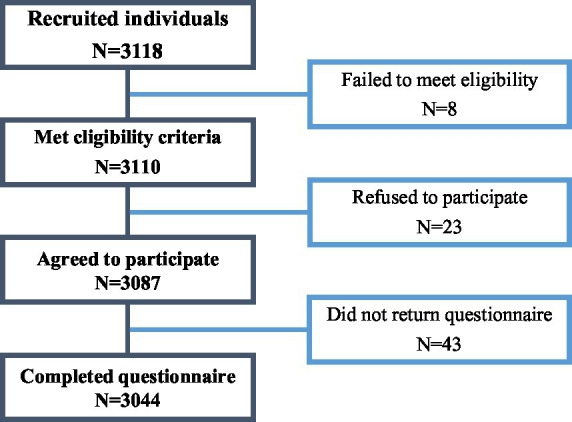
Participants selection flow diagram.

**Figure 2:**
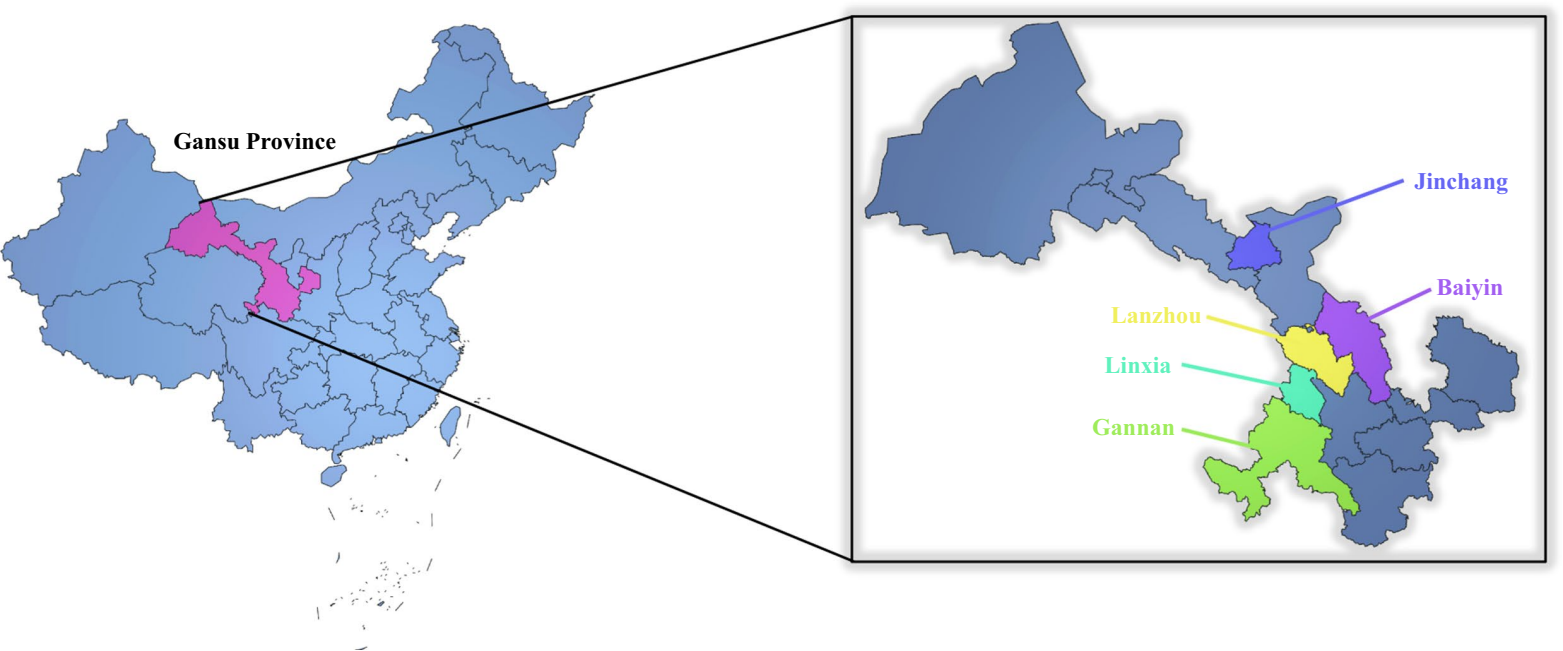
The geographical location of 5 cities in this study.

This prospective cross-sectional study systematically evaluates the prevalence and risk factors of suspected scoliosis among middle school students aged 10 to 17 from five cities in Gansu Province, China (Baiyin, Jinchang, Lanzhou, Linxia Hui Autonomous Prefecture, and Gannan Tibetan Autonomous Prefecture) (Figure 1). The study was conducted from April to July 2022. Ethical approval for this study was obtained from the Ethics Committee of Gansu Provincial Hospital (No. 2022-036-01). Parental or guardian consent was obtained before students participated in the survey. The study adhered strictly to the guidelines of Strengthening the Reporting of Observational Studies in Epidemiology (STROBE) for cross-sectional studies, as shown in Figure 2, which outlines the participant selection process.

### Data collection

We utilized a paper-based survey questionnaire developed by our team to gather information on various variables. Surveyors conducted face-to-face interviews with students to collect the questionnaire data. This information included demographic data, altitude of habitation, daily electronic device usage duration, sleep posture, family medical history, physical activity, dietary habits, sitting posture for study, daily sitting time for study, and bodily pain (neck or shoulder or low back pain). Furthermore, measurements of BMI, assessment of shoulder symmetry, evaluation of bilateral scapular symmetry, examination of waist symmetry, and determination of pelvic tilt were conducted by orthopedic physicians who have received specialized training.

### Screening of spinal curvature abnormality

Screening for suspected scoliosis was conducted by well-trained doctors specializing in pediatric spinal surgery at Gansu Provincial Hospital of Traditional Chinese Medicine. The assessment methods of suspected scoliosis involve three steps (17): visual inspection, adams forward bend test, and trunk rotation angle measurement (Supplementary Figure S1).

The screening procedure adheres to the International Society on Scoliosis Orthopedic and Rehabilitation Treatment (SOSORT) consensus document on scoliosis school screening (18). All individuals underwent screening are subjected to visual inspection, Adams forward bend test, and trunk rotation angle (ATR) measurement. Firstly, the screening subject removed the upper garment, exposing the area from the shoulders to the hips (males with bare upper body, females with tight-fitting vest). While the subject stood with feet together in a natural upright position, the examiner carefully observed and documented the levelness of the shoulders, the symmetry of the scapulae, the symmetry of the waist, and the presence of any pelvic tilt. We assessed pelvic tilt clinically through visual inspection, observing asymmetry in the coronal plane. Any affirmative response indicated a positive result in visual inspection. Secondly, the screening subject maintained the state of exposing the shoulders to the hip area with the upper garment removed. Bent forward at the waist until the back was parallel to the ground, feet together, arms naturally hanging, knees straight, palms facing each other. The examiner carefully observed and documented various aspects, including the symmetry of the spine, alignment of the shoulders parallel to the ground, symmetry of the scapulae, parallelism of the hips to the ground, alignment of the head with the midline of the pelvis, and the presence of any protruding ribs. Any affirmative response indicated a positive result in the Adams Forward Bend Test. Thirdly, performed ATR assessment on individuals who exhibited a positive outcome in the Adams forward bend test. The doctor held the scoliosis screening tool, keeping the device in direct contact with the subject’s back. The doctor scanned down the spine from the neck, reading angles when passing through the upper thoracic vertebrae, mid-thoracic vertebrae, and thoracolumbar vertebrae. Alternatively, the doctor could press a button on the device to record the angles at these three locations. The maximum angle was taken as the screening result. The main reasons for choosing ATR over X-rays for initial screening are as follows: Firstly, the portability challenges of large X-ray instruments and their cumbersome handling posed significant obstacles to conducting this large-scale screening across multiple schools and districts. Secondly, the high sensitivity and specificity of ATR measurements in screening make them an effective alternative to X-rays, especially in resource-limited settings, according to the recommendations of the USPSTF (United States Preventive Services Task Force) (17). Thirdly, we took into account the need to reduce radiation exposure, lower the cost of screening, and increase the feasibility and accessibility of screening. Especially in adolescents, repeated X-ray examinations may increase the risk of breast cancer (19, 20). In addition, there is a correlation between the ATR angle and the Cobb angle measured in X-rays (21). When ATR > 5°, the diagnostic accuracy of scoliosis is higher (22). Therefore, we used the angular trunk rotation (ATR) measure as the basis for the determination of suspected scoliosis assessment. The electronic spine measurement instrument we used during screening is model A24837H4016310, produced by Wuhan Edmond Technology Co., Ltd. in China.

### Data management and statistical analysis

In our study, all statistical analyses were performed using R software, version 4.2.2, along with MSTATA software.^1^ Graphs were created using GraphPad Prism 8.0 (San Diego, United States, GraphPad Software) and Microsoft Office PowerPoint 2019. Continuous variable data were presented as mean ± standard deviation. Categorical variables were described as counts (percentages). Continuous variables were compared using independent sample *t*-tests or non-parametric tests, while categorical variables were compared using Pearson’s chi-square test or Fisher’s exact test. *p* value <0.05 was considered statistically significant.

Logistic regression analysis was used to identify factors related to adolescent suspected scoliosis. Initially, univariate logistic regression analysis was employed to screen factors potentially associated with the outcome variable. The variables included in the univariate logistic regression analysis were as follows: age, BMI, gender, race, city, altitude of, sleeping posture, sleep time, daily electronic device usage duration, sitting posture for study, family history of suspected scoliosis, daily sitting time for study, exercise regularly, staple food preference, fruit consumption, neck or shoulder or low back pain, shoulders of unequal height.

Subsequently, a backward stepwise regression method (using the stepAIC function in the MASS package in R) was employed to select the best variable combination from the univariate logistic regression analysis for inclusion in the multivariate logistic regression analysis. This aimed to identify independent risk factors for adolescent suspected scoliosis. The selection process was based on minimizing the AIC value, which measures the goodness-of-fit and complexity of the model. The backward stepwise regression procedure involved starting with a model that included all potential variables, and then iteratively removing variables to find the optimal combination that minimized the AIC value. This method provided insights into the complex relationships among variables while optimizing model selection based on the AIC criterion using a backward elimination approach. The odds ratio (OR) and its 95% confidence interval (CI) were presented.

## Results

### Summary of suspected scoliosis screening

A total of 3,044 adolescents aged 10–17 were included in the final analysis, with 1,555 males (51.08%) and 1,489 females (48.92%). The overall prevalence of suspected scoliosis was 5.68%. Age, gender, race, sleep position, sleep duration, daily electronic device usage duration, sitting study posture, regular exercise, daily study time, staple food preference, fruit consumption, and neck or shoulder, or low back pain were balanced between the two groups. However, compared to the healthy group (*N* = 2,871), adolescents in the scoliosis group (*N* = 173) had lower BMI levels (*p* = 0.033) and a higher family history (17.34%, *p* < 0.001). The prevalence of scoliosis in adolescents living at lower altitudes (1,400–1,600 m) was almost twice that of those at higher altitudes (1,600–3,321 m) (*p* < 0.001). Table 1 showed the demographic characteristics of the participants.

**Table 1:** Patient demographics and baseline characteristics.

Characteristic	Total, *n* (%)	Healthy group, *N* = 2,871^a^	Scoliosis group, *N* = 173^a^	*p*-value^b^
**Age (year, mean (SD))**	14.91 ± 1.42	14.90 ± 1.42	15.09 ± 1.25	0.135
**BMI (kg/m** ^**2**^ **, mean (SD))**	20.14 ± 4.84	20.2 ± 4.9	18.9 ± 3.8	<0.001
**Gender**				0.710
Male	1,555 (5.53%)	1,469 (51.17%)	86 (49.71%)	
Female	1,489 (5.84%)	1,402 (48.83%)	87 (50.29%)	
**Race**				0.640
Han	1881 (5.52%)	1,777 (61.89%)	104 (60.12%)	
Minority nationality	1,163 (5.93%)	1,094 (38.11%)	69 (39.88%)	
**City**				<0.001
Baiyin	380 (0.53%)	378 (13.166%)	2 (1.156%)	
Gannan	805 (5.22%)	763 (26.576%)	42 (24.277%)	
Jinchang	499 (5.81)	470 (16.371%)	29 (16.763%)	
Lanzhou	470 (10%)	423 (14.734%)	47 (27.168%)	
Linxia	890 (5.96%)	837 (29.154%)	53 (30.636%)	
**Altitude of habitation (m)**				<0.001
1,400–1,600	988 (8.09%)	908 (31.63%)	80 (46.24%)	
1,600–2000	1,041 (4.23%)	997 (34.73%)	44 (25.43%)	
2000–3,321	1,015 (4.83)	966 (33.65%)	49 (28.32%)	
**Sleeping posture**				0.304
Lateral position predominates	2027 (5.33%)	1,919 (66.84%)	108 (62.43%)	
Prone position predominates	167 (4.79%)	159 (5.54%)	8 (4.62%)	
Supine position predominates	850 (6.71%)	793 (27.62%)	57 (32.95%)	
**Sleep duration**				0.477
≤8 h/day	2063 (5.48%)	1,950 (67.92%)	113 (65.32%)	
>8 h/day	981 (6.12)	921 (32.08%)	60 (34.68%)	
**Daily electronic device usage duration**				0.633
≤1 h/day	819 (5.49%)	774 (26.96%)	45 (26.01%)	
1 ~ 2 h/day	1,405 (5.41%)	1,329 (46.29%)	76 (43.93%)	
>2 h/day	820 (6.34%)	768 (26.75%)	52 (30.06%)	
**Sitting posture for study**				0.332
Sitting upright	1793 (6.02%)	1,685 (58.69%)	108 (62.43%)	
Bad sitting posture	1,251 (5.19%)	1,186 (41.31%)	65 (37.57%)	
**Family medical history of scoliosis**				0.033
No	2,673 (5.35%)	2,530 (88.12%)	143 (82.66%)	
Yes	371 (8.09%)	341 (11.88%)	30 (17.34%)	
**Sitting time for study (h/day)**				0.641
≤12 h/day	2,349 (5.58%)	2,218 (77.26%)	131 (75.72%)	
>12 h/day	695 (6.04%)	653 (22.74%)	42 (24.28%)	
**Exercise regularly**				0.416
Yes	2,681 (5.56%)	2,532 (88.19%)	149 (86.13%)	
No	363 (6.61%)	339 (11.81%)	24 (13.87%)	
**Staple food preference**				0.459
Rice	943 (6.36%)	883 (30.76%)	60 (34.68%)	
Wheaten	723 (5.81%)	681 (23.72%)	42 (24.28%)	
Both	1,378 (5.15%)	1,307 (45.52%)	71 (41.04%)	
**Fruit consumption**				0.901
>1 times/day	1,640 (5.73%)	1,546 (53.85%)	94 (54.34%)	
≤1 times/day	1,404 (5.63%)	1,325 (46.15%)	79 (45.66%)	
**Neck or shoulder or low back pain**				0.260
No	537 (6.70%)	501 (17.45%)	36 (20.81%)	
Yes	2,507 (5.46%)	2,370 (82.55%)	137 (79.19%)	
**Shoulders of unequal height**				0.011
No	2011 (4.92%)	1,912 (66.60%)	99 (57.23%)	
Yes	1,033 (7.16%)	959 (33.40%)	74 (42.77%)	

a Median (IQR); *n* (%).

b Wilcoxon rank sum test; Pearson’s Chi-squared test.

BMI, body mass index.

### Prevalence of suspected scoliosis stratified by age and city

**Figure 3:**
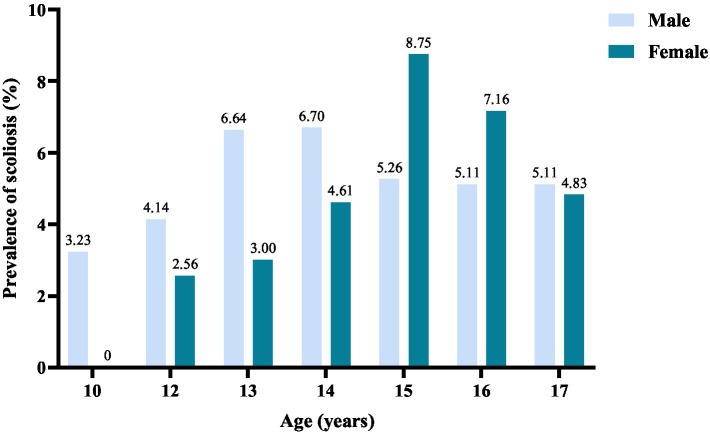
Prevalence of scoliosis in adolescents of different ages.

Overall, the prevalence of suspected scoliosis in males and females was similar, with rates of 5.53 and 5.84%, respectively (*p* = 0.710). The prevalence of suspected scoliosis reaches its zenith with advancing age in both males and females, subsequently tapering off gradually. The peak prevalence for males is at 14 years (6.70%), while for females, it is at 15 years (8.75%) (Figure 3).

**Figure 4:**
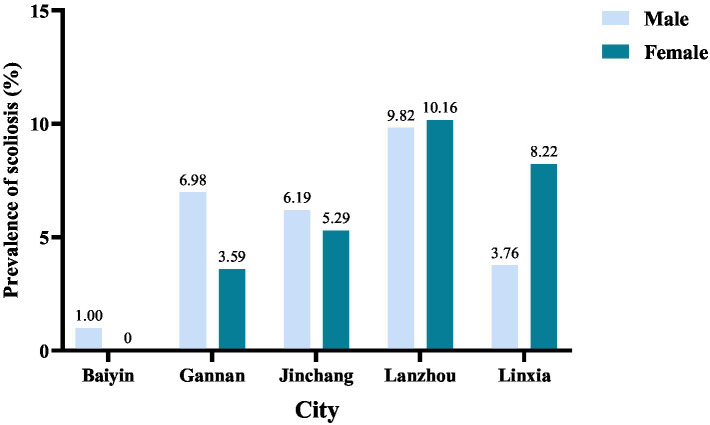
Prevalence of scoliosis in adolescents of 5 cities.

In the comparative analysis of suspected scoliosis prevalence among various cities, the incidence rates are relatively higher for boy in Gannan Autonomous Prefecture (6.98%) and Jinchang City (6.19%). Meanwhile, the prevalence is notably higher among girl in Linxia Autonomous Prefecture (8.22%). Notably, in the five cities, Lanzhou City exhibits the highest prevalence rates for both boy and girl. The prevalence is 9.82% for both and 10.16% for girl (Figure 4).

### Results of univariate logistic regression analysis

**Figure 5:**
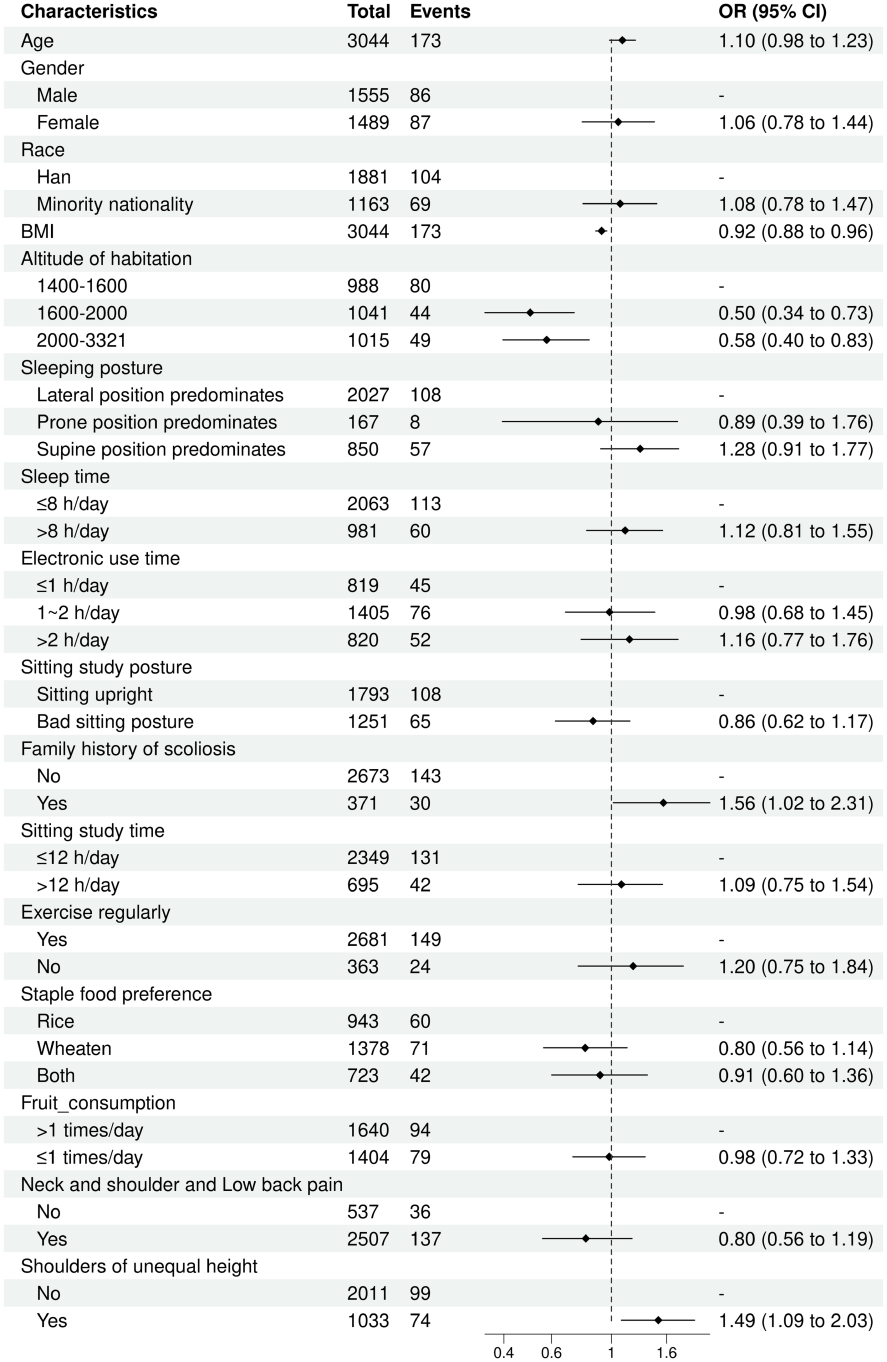
Associated factors with scoliosis by univariate logistic regression analysis.

The results of univariate logistic regression analysis presented that BMI (OR = 0.92, 95% CI: 0.88–0.96), altitude of habitation (1,600 m-2000 m) (OR = 0.50, 95% CI: 0.34–0.73), altitude of habitation (2000 m-3321 m) (OR = 0.58, 95% CI: 0.40–0.83), family medical history (OR = 1.56, 95% CI: 1.02–2.31), and shoulders of unequal height (OR = 1.49, 95% CI: 1.09–2.03) were significantly correlated with suspected scoliosis (Figure 5).

### Results of multivariate logistic regression analysis

**Figure 6:**
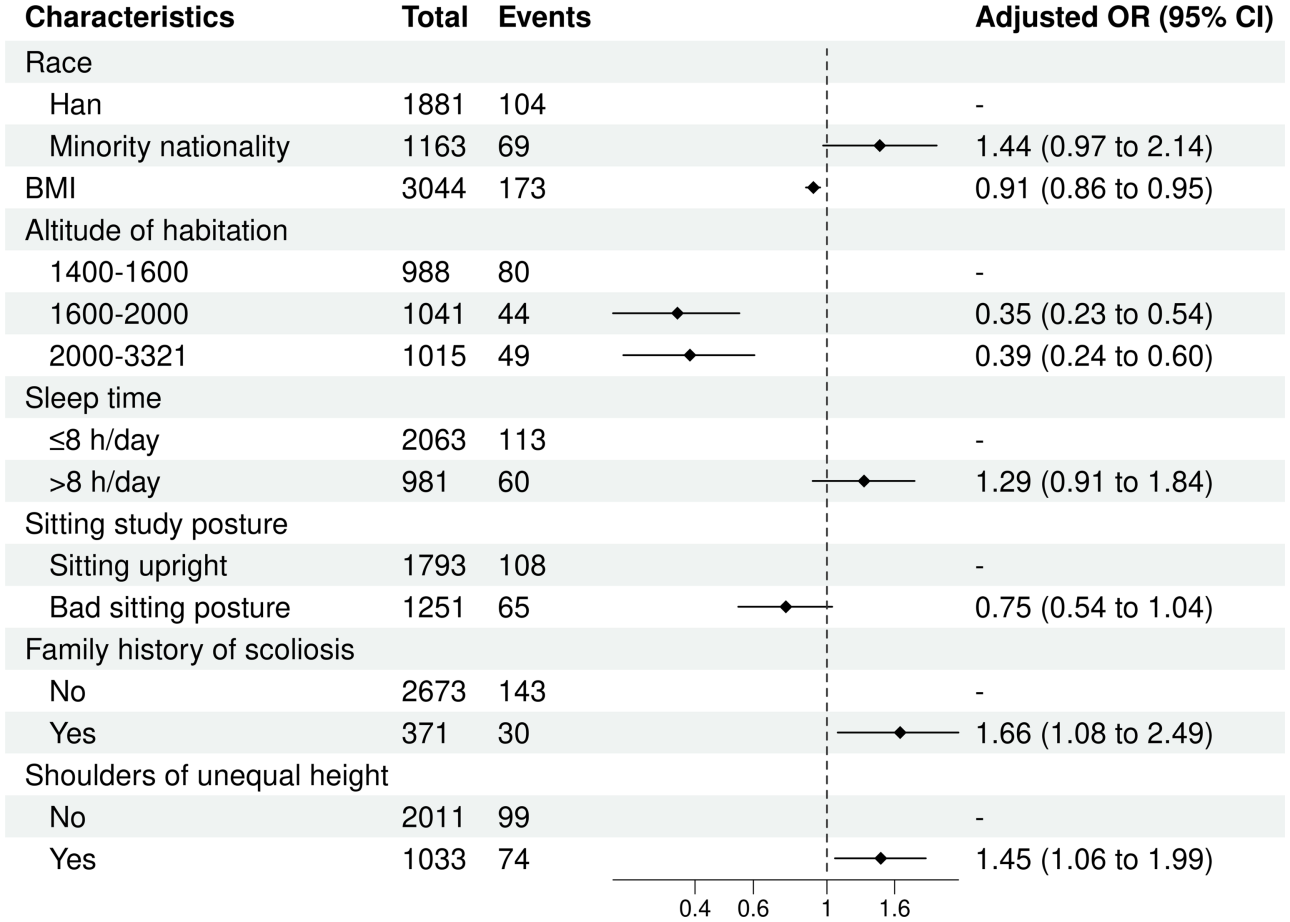
Associated factors with scoliosis by multivariate logistic regression analysis.

The results of the backward stepwise regression show that race, BMI, altitude of habitation, sleep duration, sitting posture for study, family medical history, and shoulders of unequal height were the optimal variable combinations. Hence, these variables were included in the multivariate logistic regression analysis. The multivariate logistic regression analysis indicated that BMI (OR = 0.91, 95% CI: 0.86–0.95), altitude of habitation (1,600 m-2000 m) (OR = 0.35, 95% CI: 0.23–0.54), altitude of habitation (2000 m-3321 m) (OR = 0.39, 95% CI: 0.24–0.60), family medical history (OR = 1.66, 95% CI: 1.08–2.49), and shoulders of unequal height (OR = 1.45, 95% CI: 1.06–1.99) were independently associated with suspected scoliosis (Figure 6).

## Discussion

This cross-sectional study systematically investigated the prevalence of adolescent suspected scoliosis and associated risk factors in five cities in Gansu Province, China. We found that the BMI, altitude of habitation, family medical history, and shoulders of unequal height were independent influencing factors for suspected scoliosis. The overall prevalence of suspected scoliosis in adolescents in Gansu Province, China, was 5.68%. Additionally, the peak ages for suspected scoliosis prevalence in males and females were 14 and 15 years, respectively.

In this study, the overall prevalence of suspected scoliosis, at 5.68%, exhibited a lower rate compared to Japan (7.6%) (23), yet surpassed that of Jammu and Kashmir in India (0.61%) (24), and Singapore (1.37 and 2.22%) (25), among other regions. In comparison to related studies in mainland China, our result was lower than in the Shanghai region (6.9%) (26) and higher than in Dali, Yunnan province (2.37%) (16), and the Qinghai-Tibet Plateau region (3.69%) (27). Our study primarily focused on adolescents aged 10–17, excluding elementary school students below 10 years old. Therefore, we lacked screening data related to early-onset suspected scoliosis (under 10 years old), leading to discrepancies with epidemiological survey data from domestic and international sources. Moreover, environmental and climatic factors in different regions may constitute significant sources of heterogeneity in the prevalence of spinal curvature.

Considering the peak age of onset, we found that 14 years old was the peak age for suspected scoliosis in male students in Gansu Province, while 15 years old was the peak for female students. This aligns with the results of Huang et al.’s study (26). Additionally, Penha et al.’s research (4) in São Paulo, Brazil, reported that scoliosis typically occurs in late childhood, with over 4% of adolescents aged 11–17 showing spinal deformities. This correlation may be attributed to growth patterns, coinciding with the accelerated developmental phase of the spinal column observed in children aged 10–15 years (28, 29).

The 3,044 students were distributed across five different cities. According to our research findings, there was a significant difference in the prevalence of suspected scoliosis among the five cities. Baiyin City, Gannan Autonomous Prefecture, and Jinchang City had a higher prevalence rate among males. In Linxia Autonomous Prefecture, the prevalence rate was higher among females. Lanzhou City had the highest prevalence rate among the five cities, with rates of 9.82% for males and 10.16% for females. One possible explanation is that as the provincial capital of Gansu Province, Lanzhou City places significant academic pressure on students, which was notably higher than in the other four regions. Students in Lanzhou may not be receiving adequate rest and appropriate physical activity (26).

Another interesting finding was that students living at an altitude of 1,600 m-3321 m had a significantly lower prevalence of scoliosis compared to those living at an altitude of 1,400 m-1600 m. This result differed from previous research. Zhou et al. conducted a cross-sectional study on scoliosis in Xizang, China (27). They found that both the prevalence of scoliosis and the rate of surgery were highly independent of residence. For children living below 4,500 meters, the prevalence of scoliosis was 3.50%, while the prevalence of scoliosis in children living above 4,500 meters was 5.63% (27). There was no significant difference in the prevalence of idiopathic scoliosis between the two groups, so they concluded that the higher prevalence at high altitudes was mainly due to congenital scoliosis and neuromuscular scoliosis (27). This finding may be due to hypoxic conditions at high altitudes, which can disrupt embryonic development and then lead to organ defects in many systems (30). A study conducted in Tibet suggests that higher altitudes may increase the risk of delayed height growth, which could have long-term negative effects on children from birth to the age of three (31). However, most of these studies focused on extremely high-altitude areas (altitude >3,500 m), where issues such as hypoxia, economic underdevelopment, cold climate, and insufficient food diversity may negatively impact children’s musculoskeletal development and function, including vitamin D deficiency (32). In contrast, our study area is in a moderately high-altitude region (1,500 m-3500 m), where the adverse effects of hypoxia are not as pronounced as in extremely high-altitude areas. Additionally, with the implementation of China’s poverty alleviation policies, the economic level of Gansu Province has significantly improved. Children born after 2000 are essentially free from malnutrition.

Genetic factors, recognized as contributing factors to scoliosis, were evident in this study. Results from both univariate and multivariate regression analyses indicated a significant correlation between a family medical history and the presence of suspected scoliosis. There may be shared lifestyle and environmental factors between parents and children, which could also impact the spinal health of the children. This finding aligns with previous research. Cheng et al. conducted a familial study identifying a genetic component in idiopathic scoliosis (33). A cross-sectional study expounded that adolescents with spinal deformities in either parent or both parents had odds ratios for developing scoliosis of 1.46, 1.74, and 2.58, respectively, compared to those without deformities in their parents (34). Simony et al. studied 274 pairs of twins in Denmark and determined a higher concordance rate in monozygotic twins compared to dizygotic twins (35). Justice et al.’s familial study indicated a significant association between susceptibility loci on the X chromosome and the genetic risk of scoliosis (36). In a genome-wide linkage analysis of dominant transmission patterns in three large multiple IS families, Patrick Edery et al. found that scoliosis has a high degree of genetic heterogeneity, meaning that there may be different genetic factors within different families and within the same family (37). Although the genetic pattern is subject to debate, current research suggested that chromosomal abnormalities and gene loci variations lead to primary gene expression changes. Epigenetic changes induced by environmental factors further regulated gene expression. These factors collectively induced disruptions in cellular activity, ultimately contributing to the development of AIS.

BMI serves as a comprehensive indicator reflecting body size and nutritional status. Numerous studies had consistently identified low BMI as a major contributing factor to the development of scoliosis. Our study corroborated this association, indicated a significant correlation between low BMI and suspected scoliosis. Clinical research had reported that compared to healthy adolescents, those with AIS exhibit a marked reduction in BMI (38). Kim et al.’s study further suggested that slender students may have up to a fourfold increased likelihood of developing scoliosis (39). Another Chinese study found that individuals with scoliosis have higher rates of low BMI compared to healthy students (40). A Mendelian randomization study confirmed a causal relationship between genetic variations leading to low BMI and the incidence of AIS (14).

Shoulder balance is a crucial component of body posture in adolescents with AIS. This study found a significant correlation between uneven shoulders and suspected scoliosis, consistent with previous research findings. A survey by Qiu et al. (41) confirmed that the left shoulder of adolescents with AIS often elevates, accompanied by bilateral thoracic curvature and T1 tilt. Raso et al. (42) conducted X-ray and surface examinations on 20 scoliosis patients, revealing that 75% of overall deformities included asymmetry of the scapula, shoulder angles, and lumbar asymmetry. A study by Barishan et al. (43) revealed that a rib-vertebral angle crossing 4 degrees could serve as a diagnostic parameter for radiographic shoulder joint imbalance in scoliosis. These findings suggested a potential association between scoliosis patients and shoulder imbalance.

Undoubtedly, this study has some limitations. Firstly, this study is a cross-sectional study, making it unable to establish a causal relationship between risk factors and suspected scoliosis. Secondly, we did not choose to perform X-ray examination on the adolescents in this study because of the characteristics of mass screening studies and the limitation of medical resources, as well as considering the radiation effects of X-rays. Third, the ATR measurements used in this study may have a slight error in determining suspected scoliosis, and future X-ray testing of screened children with scoliosis is still needed for definitive diagnosis. Additionally, younger children were not included, and information about them was not collected due to the original design of this study, potentially introducing some minor bias.

## Conclusion

The overall prevalence of adolescent suspected scoliosis in Gansu Province, China, is 5.68%. Low BMI, residence at an altitude of 1,600 m-3321 m, family medical history, and shoulders of unequal height were independently associated with an increased prevalence. Boys at 14 years old and girls at 15 years old should pay particular attention to the occurrence of suspected scoliosis. This study provides a vital reference for formulating public health policy directions at the national level. It is recommended to promptly screen high-risk adolescents for suspected scoliosis, provide effective preventive and intervention measures, and establish an integrated health education model involving schools, families, and hospitals.

## Data availability statement

The original contributions presented in the study are included in the article/Supplementary material. Further inquiries can be directed to the corresponding author.

## Ethics statement

The studies involving humans were approved by Gansu Provincial Hospital of Traditional Chinese Medicine, Approval (No. 2022-014-01). The studies were conducted in accordance with the local legislation and institutional requirements. Written informed consent for participation in this study was provided by the participants’ legal guardians/next of kin.

## Author contributions

JH: Conceptualization, Data curation, Formal analysis, Investigation, Methodology, Software, Supervision, Validation, Visualization, Writing – original draft, Writing – review & editing. HZ: Conceptualization, Formal analysis, Investigation, Methodology, Software, Supervision, Validation, Visualization, Writing – original draft, Writing – review & editing. JiW: Conceptualization, Investigation, Methodology, Validation, Writing – original draft. LL: Conceptualization, Data curation, Investigation, Validation, Visualization, Writing – original draft. SX: Conceptualization, Data curation, Investigation, Writing – original draft. XW: Conceptualization, Investigation, Methodology, Writing – original draft. CZ: Data curation, Investigation, Writing – original draft. HW: Data curation, Investigation, Writing – original draft. SP: Conceptualization, Data curation, Investigation, Writing – original draft. XC: Data curation, Investigation, Writing – original draft. JuW: Data curation, Investigation, Writing – original draft. DT: Conceptualization, Methodology, Resources, Supervision, Validation, Writing – review & editing. JZ: Conceptualization, Data curation, Funding acquisition, Investigation, Resources, Writing – review & editing.

## Footnotes

^1^ http://www.mstata.com

## Supplementary material

SUPPLEMENTARY FIGURE 1 Forward Bend Test and Scoliometer Usage Diagram. **(A)** Initial Visual Inspection, the clinician performs an initial visual inspection of the patient’s spine while the patient stands upright with feet together. To look for any noticeable asymmetry in the shoulders, shoulder blades, and hips, which might indicate scoliosis. **(B)** Forward Bend Test, the patient stands with feet together and bends forward at the waist with arms hanging down and palms touching. To allow the clinician to perform a second visual inspection of the spine in this position, which can reveal spinal abnormalities not visible when standing upright. **(C)** Scoliometer Measurement (Normal), the scoliometer is placed on the patient's back while they are in the forward bend position. To measure the angle of trunk rotation. A low angle (within normal range) indicates no significant spinal curvature. **(D)** Scoliometer Measurement (Abnormal), the scoliometer is used in the same manner as in **C**, but here it shows an increased angle of trunk rotation. An angle of trunk rotation between 5° and 7° or higher suggests scoliosis.
