# Supplementary figures and images for: Evaluation of the prevalence of adolescent scoliosis and its associated factors in Gansu Province, China: a cross-sectional study
Source: Front Public Health. 2024 Jul 30;12:1381773. doi: 10.3389/fpubh.2024.1381773 (PMC11319255; doi:10.3389/fpubh.2024.1381773)

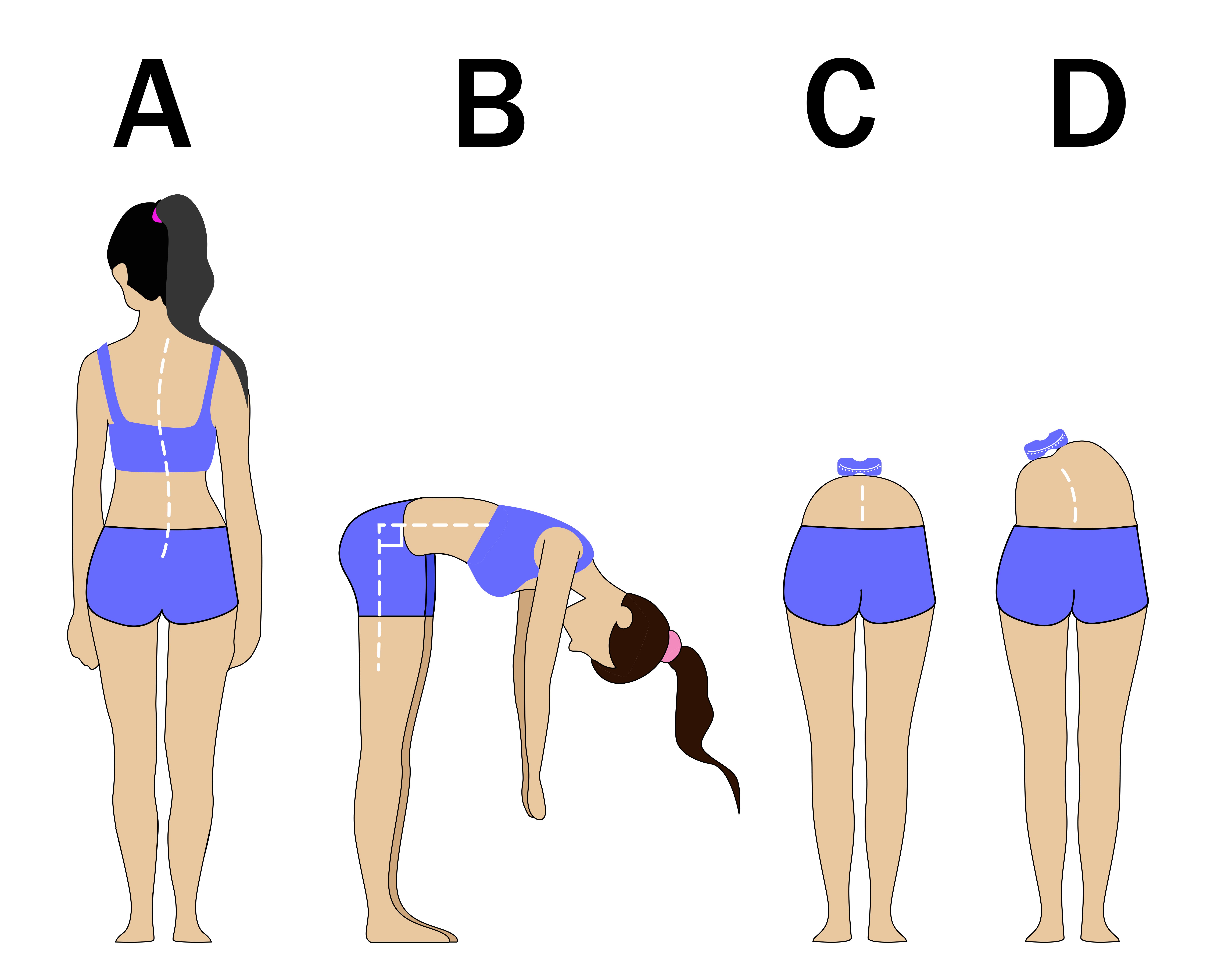

Supplement: SUPPLEMENTARY FIGURE 1 — Forward Bend Test and Scoliometer Usage Diagram. (A) Initial Visual Inspection, the clinician performs an initial visual inspection of the patient’s spine while the patient stands upright with feet together. To look for any noticeable asymmetry in the shoulders, shoulder blades, and hips, which might indicate scoliosis. (B) Forward Bend Test, the patient stands with feet together and bends forward at the waist with arms hanging down and palms touching. To allow the clinician to perform a second visual inspection of the spine in this position, which can reveal spinal abnormalities not visible when standing upright. (C) Scoliometer Measurement (Normal), the scoliometer is placed on the patient's back while they are in the forward bend position. To measure the angle of trunk rotation. A low angle (within normal range) indicates no significant spinal curvature. (D) Scoliometer Measurement (Abnormal), the scoliometer is used in the same manner as in C, but here it shows an increased angle of trunk rotation. An angle of trunk rotation between 5° and 7° or higher suggests scoliosis. [file Image_1.png]
